# An Explainable AI Framework for Continuous Monitoring, Risk Stratification, and Clinical Decision Support in Primary Biliary Cholangitis: Protocol for a Multiphase Development and Validation Study

**DOI:** 10.2196/89279

**Published:** 2026-06-24

**Authors:** Basile Njei, Ulrick Sidney Kanmounye

**Affiliations:** 1 Engelhardt School of Global Health and Bioethics, Euclid University, Avenue de France, Campus ENAM, Bangui, BP 157, Central African Republic, +236 21 61 59 2; 2 Association of Future African Neurosurgeons, Yaounde, Cameroon

**Keywords:** artificial intelligence, AI, primary biliary cholangitis, randomized controlled trials, clinical decision support, portal hypertension, explainable artificial intelligence, patient-reported outcomes

## Abstract

**Background:**

Primary biliary cholangitis (PBC) management remains limited by reliance on static biochemical markers, fragmented assessment of symptom burden, and inadequate noninvasive risk stratification for clinically significant portal hypertension (CSPH). Existing tools fail to integrate longitudinal laboratory trends, elastography, and patient-reported outcomes, and translation of risk assessment into guideline-concordant clinical action remains inconsistent.

**Objective:**

This study aims to develop, validate, and pilot an explainable artificial intelligence (AI) framework—AIm-PBC—for continuous disease monitoring, early prediction of CSPH complications, and delivery of guideline-based clinical decision support in patients with PBC.

**Methods:**

We will conduct a multiphase study combining retrospective cohort analysis, prospective observational data collection, and implementation evaluation. At least 600 adults with confirmed PBC will be enrolled across academic hepatology centers. The AI framework will integrate longitudinal biochemical markers, noninvasive elastography metrics, and high-frequency patient-reported outcomes to generate an interpretable disease activity index and predict CSPH-related complications using gradient-boosted models with Shapley additive explanations. Outputs will be deployed via a SMART-on-FHIR (Substitutable Medical Applications, Reusable Technologies on Fast Healthcare Interoperability Resources)–enabled electronic health record clinical decision support tool. Implementation will be evaluated using a randomized crossover simulation followed by a pragmatic pilot assessing usability, cognitive load, clinician adherence, and feasibility.

**Results:**

The primary outcomes include calibration and responsiveness of the disease activity index; discriminatory performance of the CSPH prediction model compared with established criteria; and effectiveness of the decision support tool measured via improvements in guideline-concordant care, usability scores, and clinician cognitive workload. Secondary outcomes include fairness metrics and workflow efficiency.

**Conclusions:**

This protocol outlines a scalable, explainable AI framework designed to bridge gaps between disease monitoring, risk prediction, and clinical action in PBC. If successful, AIm-PBC may enhance early complication detection, improve symptom management, and support equitable, evidence-based care delivery in routine hepatology practice.

## Introduction

### Background

Despite advances in primary biliary cholangitis (PBC) therapy, significant gaps persist in monitoring disease activity, predicting complications, and delivering guideline-concordant care [1-4]. Current management strategies rely heavily on biochemical markers such as alkaline phosphatase (ALP) and bilirubin, which often fail to capture dynamic symptom fluctuations or early progression toward clinically significant portal hypertension (CSPH) [5,6]. This limitation delays timely intervention and contributes to morbidity [7].

Risk stratification for portal hypertension, a complication of PBC, remains particularly challenging [8]. Invasive procedures such as endoscopy are frequently used for variceal screening, yet these approaches are resource intensive and carry procedural risks [2,4]. Noninvasive surrogates, including liver stiffness measurement (LSM) and spleen stiffness measurement (SSM), have demonstrated promise in predicting CSPH and reducing unnecessary endoscopies [2,4,9]. However, these modalities are rarely integrated with longitudinal biochemical data or patient-reported outcomes (PROs), leaving clinicians without a comprehensive, real-time view of disease trajectory [10]. Even when risk is identified, translating insights into action remains challenging. Clinicians face cognitive overload and workflow fragmentation, leading to missed opportunities for guideline-based interventions such as pruritus step therapy, bone health management, and CSPH screening [3].

Symptom burden represents another critical dimension of PBC care. Validated instruments such as the Worst Itch Numeric Rating Scale (WI-NRS) and PBC-40 provide reliable measures of symptom severity [10,11]. Similarly to risk stratification tools, symptom burden tools are underused in routine practice and seldom incorporated into predictive models.

### Proposed Solution

Artificial intelligence (AI) offers an opportunity to address these gaps by integrating heterogeneous data streams, including biochemical markers, elastography metrics, and PROs, into dynamic, explainable models capable of continuous monitoring and risk prediction [12-14]. By developing an agentic AI system that integrates longitudinal biochemical data, elastography metrics, and high-frequency PROs, we aim to provide clinicians with a dynamic, explainable disease activity index. This approach will enable proactive management of symptoms and early identification of nonresponders [15], reducing morbidity and improving patient-centered outcomes. Our multi-signal predictive model will integrate elastography, platelet count, biochemical response, and comorbidity profiles to accurately identify patients at high risk of early CSPH complications. This composite approach promises to reduce unnecessary endoscopies while ensuring timely prophylaxis for those at greatest risk. By embedding explainable AI outputs into an electronic health record (EHR)–integrated clinical decision support (CDS) tool, we aim to bridge the gap between prediction and practice. This prototype will feature interpretable risk cards and dual-threshold triage cues, ensuring usability and trust while promoting equity across diverse clinical settings. While machine learning algorithms have shown promise in chronic liver disease, most operate as “black boxes,” limiting clinician trust and adoption. Consequently, we aim to use Shapley additive explanations (SHAP) combined with dual-threshold triage strategies to enhance transparency and usability.

### Study Aim

**Figure 1.**
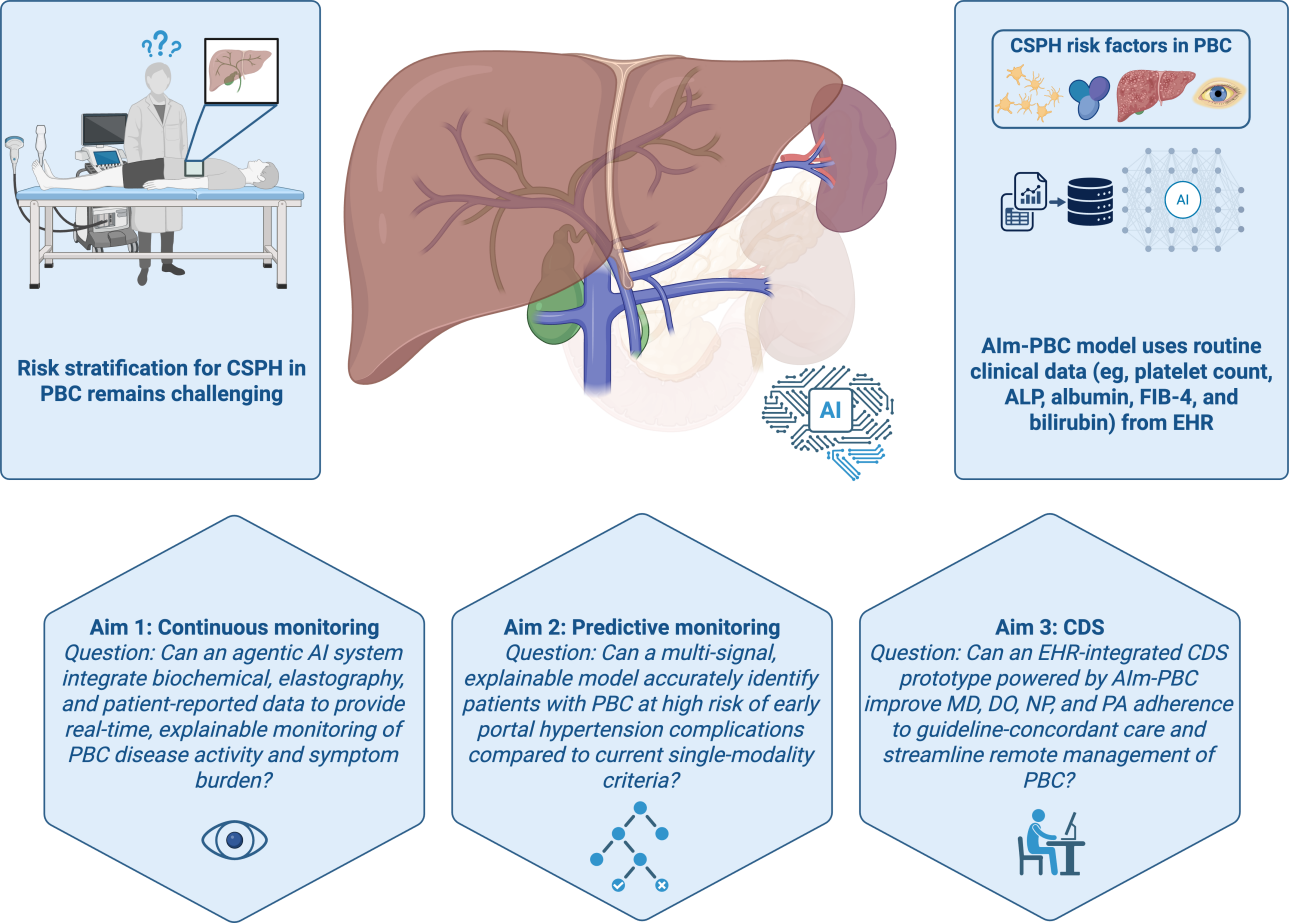
Conceptual framework for the explainable artificial intelligence (AI) system for symptom and disease monitoring, early portal hypertension prediction, and remote clinical decision support (CDS) in primary biliary cholangitis (PBC). ALP: alkaline phosphatase; AQFIB-4: Fibrosis-4 index for liver fibrosis; CSPH: clinically significant portal hypertension; DO: doctor of osteopathic medicine; EHR: electronic health record; MD: doctor of medicine; NP: nurse practitioner; PA: physician assistant.

This protocol describes a multiphase study designed to develop, validate, and pilot this explainable AI framework for PBC management. The proposed framework, named AIm-PBC system, will integrate longitudinal multi-signal data to (1) provide continuous, interpretable assessment of disease activity and symptom burden; (2) predict CSPH complications using a composite noninvasive model; and (3) operationalize guideline-concordant care through an EHR-integrated CDS prototype (Figure 1). By leveraging implementation science principles, this study aims to ensure feasibility, usability, and equity across diverse clinical environments.

### Hypothesis

We hypothesize that this integrated, explainable AI approach will outperform conventional monitoring strategies, improve risk stratification for CSPH, and enhance clinician adherence to evidence-based care pathways, ultimately reducing symptom burden and preventing complications in patients with PBC.

## Methods

### Study Design

This multiphase study will combine retrospective cohort analysis, prospective observational data collection, and implementation science evaluation using a randomized crossover simulation followed by a pragmatic pilot. Its aims address critical unmet needs in PBC care by leveraging explainable AI to integrate multimodal data streams, predict complications, and operationalize evidence-based management. The study consists of 3 sequential phases: retrospective model development, prospective simulation testing, and real-world implementation. A summary of the objectives, data sources, participants, and outcomes for each phase is provided in Table 1.

**Table 1.** Overview of study phases, objectives, data sources, and outcomes.

Phase	Study type	Objective	Data sources	Participants	Primary outcomes
Phase 1: model development and retrospective validation	Retrospective analysis	To develop and retrospectively validate the AI^a^-based predictive model for PBC^b^-associated significant fibrosis using existing clinical datasets	Retrospective EHR^c^ datasets and previously collected clinical variables	Adult patients with MASLD and available fibrosis assessment data	Model discrimination and calibration for predicting significant fibrosis (eg, AUROC^d^, sensitivity, and specificity)
Phase 2: prospective simulation and workflow testing	Prospective simulation	To evaluate how the AI system performs within simulated primary care workflows and refine decision thresholds and outputs	Prospective simulated clinical cases and structured datasets reflecting real-world patient characteristics	Simulated patient scenarios evaluated by clinicians using the AI decision support system	Diagnostic accuracy, workflow integration feasibility, and clinician usability metrics
Phase 3: real-world clinical implementation	Prospective real-world implementation	To assess the clinical performance and implementation feasibility of the AI decision support system in routine care	Real-world clinical data collected during system deployment in participating clinical settings	Adult patients undergoing evaluation for MASLD in real clinical practice	Detection rate of significant fibrosis, clinical decision impact, and implementation feasibility metrics

a AI: artificial intelligence.

b PBC: primary biliary cholangitis.

c EHR: electronic health record.

d AUROC: area under the receiver operating characteristic curve.

### Study Setting

The study will be conducted across multiple hepatology clinics affiliated with academic medical centers in the United States. The CDS pilot will be implemented within EHR environments supporting SMART-on-FHIR (Substitutable Medical Applications, Reusable Technologies on Fast Healthcare Interoperability Resources) integration.

### Eligibility Criteria

Adults aged 18 years or older with a confirmed diagnosis of PBC according to American Association for the Study of Liver Diseases criteria (ie, defined as elevated ALP of ≥1.5 times the upper limit of normal in combination with a positive antimitochondrial antibody or disease-specific antinuclear antibodies such as sp100 or gp210) [2]. For individuals who are seronegative, histological confirmation through liver biopsy will be mandatory. All participants must have been receiving standard therapeutic dosing of ursodeoxycholic acid for a minimum of 3 months prior to enrollment, defined as 13 to 15 mg per kilogram of body weight per day administered in 2 or 3 divided doses [2]. Patients who are on second-line therapies, including farnesoid X receptor agonists or peroxisome proliferator–activated receptor agonists, will also be eligible, and therapy class will be recorded as a covariate. Additionally, baseline elastography data, including LSM and SSM, must be available within 6 months of enrollment.

Individuals will be excluded if they have previously undergone liver transplantation or are currently listed for transplantation or if they present with decompensated cirrhosis at baseline, defined as Child-Pugh class B or C [16]. Other exclusion criteria include active malignancy, uncontrolled systemic illness, pregnancy, or inability to provide informed consent. Patients with significant alcohol consumption within 6 months of enrollment (ie, more than 20 grams per day for female individuals or more than 30 grams per day for male individuals or an Alcohol Use Disorders Identification Test–Consumption score of 4 or higher) [17] will not be eligible. Additional exclusions are coexisting chronic liver diseases such as chronic viral hepatitis, hemochromatosis, Wilson disease, or alpha-1 antitrypsin deficiency, as well as active or suspected drug-induced liver injury within the prior 6 months, including drug-induced autoimmune hepatitis (AIH). Any condition deemed by the investigators to compromise participant safety or data integrity will also result in exclusion. A summary of inclusion and exclusion criteria is provided in Textbox 1.

To enable subgroup analyses, participants will be stratified by the presence of AIH overlap, defined using the Paris criteria [18], and by the presence of metabolic dysfunction–associated steatohepatitis (MASH), identified through structured chart abstraction and imaging or laboratory features [19-21]. Stratification will also occur based on biochemical response status at 12 months following initiation of ursodeoxycholic acid therapy, categorized using established UK-PBC and GLOBE risk scores [22,23].

Textbox 1. **Inclusion and exclusion criteria.**

**Inclusion criteria**

- Adults aged ≥18 y
- Confirmed diagnosis of primary biliary cholangitis according to American Association for the Study of Liver Diseases criteria, defined as elevated alkaline phosphatase of ≥1.5 times the upper limit of normal with positive antimitochondrial antibody or disease-specific antinuclear antibodies such as sp100 or gp210 [2]
- For seronegative individuals, histological confirmation via liver biopsy
- Receiving standard therapeutic dosing of ursodeoxycholic acid for ≥3 mo before enrollment, defined as 13 to 15 mg per kilogram per day in 2 or 3 divided doses [2]
- Patients receiving second-line therapies, including farnesoid X receptor agonists or peroxisome proliferator–activated receptor agonists, with therapy class recorded as a covariate
- Baseline elastography data, including liver stiffness measurement and spleen stiffness measurement, available within 6 mo of enrollment

**Exclusion criteria**

- Prior liver transplantation or currently listed for liver transplantation
- Decompensated cirrhosis at baseline, defined as Child-Pugh class B or C [16]
- Active malignancy, uncontrolled systemic illness, pregnancy, or inability to provide informed consent
- Significant alcohol consumption within 6 mo of enrollment, defined as >20 g per day for female individuals or >30 g per day for male individuals or Alcohol Use Disorders Identification Test–Consumption score of ≥4 [17]
- Coexisting chronic liver diseases, including chronic viral hepatitis, hemochromatosis, Wilson disease, or alpha-1 antitrypsin deficiency
- Active or suspected drug-induced liver injury within the prior 6 mo, including drug-induced autoimmune hepatitis
- Any condition deemed by the investigators to compromise participant safety or data integrity

### Interventions

The intervention consists of the development and deployment of the AIm-PBC framework across 3 sequential phases. The first phase focuses on building an agentic AI system capable of continuous monitoring of disease activity and symptom burden. This system will integrate multiple data streams, including monthly biochemical markers, periodic elastography measurements such as LSM and SSM, and high-frequency PROs collected through validated instruments such as the WI-NRS and the PBC-40. The agent will autonomously orchestrate data ingestion, normalization, and analysis to generate an explainable disease activity index supported by SHAP-based visualizations to ensure interpretability for clinicians.

The second phase involves the creation and validation of a multi-signal predictive model for early identification of portal hypertension complications. This model will combine elastography metrics, platelet count, biochemical response trajectories, and comorbidity profiles, including AIH and MASH overlap. By leveraging gradient-boosted ensemble algorithms and explainability frameworks, the model aims to outperform existing single-modality rules and consensus criteria such as Baveno VII [24], providing a noninvasive, accurate method for triaging endoscopy referrals and initiating prophylactic interventions.

The third phase operationalizes these insights through an EHR-integrated CDS prototype. Built within a SMART-on-FHIR architecture, the CDS tool will present clinicians with interpretable risk cards; dual-threshold triage cues; and guideline-based order sets for interventions such as pruritus step therapy, bone health management, and endoscopy referral. This phase includes a randomized crossover simulation to evaluate usability, decision accuracy, and cognitive load, followed by a pragmatic pilot in hepatology clinics to assess feasibility and effectiveness in real-world workflows. Collectively, these interventions aim to transform PBC care by embedding explainable AI into routine practice, improving monitoring, risk prediction, and adherence to evidence-based management strategies.

### Outcomes

The primary outcome for the first aim will be the performance of the AIm-PBC disease activity index compared to conventional monitoring strategies. This will be assessed through its calibration against established risk scores such as UK-PBC and GLOBE [22,23], as well as its responsiveness to clinically meaningful changes in disease activity. The ability of the integrated symptom indexes to correlate with biochemical progression or regression will also be evaluated, providing insights into whether the inclusion of PROs enhances the sensitivity of disease monitoring. Explainability will be measured via the interpretability of SHAP-based visualizations and their perceived clinical utility during clinician evaluations.

For the second aim, the primary outcome will be the discriminatory ability of the multi-signal predictive model to identify patients at high risk of CSPH and early complications such as variceal hemorrhage or ascites. This will be quantified using the area under the receiver operating characteristic curve (AUROC), with additional measures of calibration and clinical utility assessed through decision curve analysis and net reclassification improvement. Comparative analyses will determine whether the composite model outperforms single-modality rules and consensus criteria such as Baveno VII, and subgroup analyses will explore performance consistency across patients with AIH overlap and MASH.

For the third aim, the primary outcome will be the effectiveness and usability of the EHR-integrated CDS prototype. Effectiveness will be measured via improvements in clinician adherence to guideline-concordant care pathways, including pruritus management, bone health interventions, and risk-based endoscopy referrals. Usability will be assessed using the System Usability Scale, with a target score of 70 or higher, and cognitive load will be evaluated using the NASA-Task Load Index. Clinician trust in the AI system will be measured through the AI trust scale, whereas decision accuracy and decision time will be compared between CDS-assisted and usual care conditions during the randomized crossover simulation. Feasibility indicators, including recruitment and completion rates, will be monitored during the pragmatic pilot phase.

Secondary outcomes will include fairness metrics to ensure equitable model performance across demographic and clinical subgroups, defined as an AUROC parity gap of no more than 5%. Additional exploratory outcomes will examine the impact of the intervention on workflow efficiency and clinician confidence in decision-making.

### Harms

This pilot implementation component is considered minimal risk for multiple reasons. First, the randomized crossover simulation will involve standardized simulated or deidentified patient cases rather than live clinical encounters. The participants in this simulation component will be health care providers who interact with an EHR interface in a controlled environment. The decision support tool will provide risk estimates and triage recommendations based on synthetic or deidentified data. As such, the simulation component is not expected to pose physical harm, diagnostic error affecting patient care, or adverse clinical outcomes. For the observational/model-development components, adults with PBC will contribute clinical, elastography, and patient-reported outcome data under institutional review board–approved procedures and standard privacy protections.

### Participant Timeline

Participants will enter the study and begin with baseline assessments, which include collection of demographic information, medical history, and initial laboratory values, as well as elastography measurements for liver and spleen stiffness. At this visit, participants will also be enrolled in the PRO system, with instructions for completing the WI-NRS and the PBC-40 questionnaires. These assessments will continue throughout the observation period to capture longitudinal symptom trajectories. The schedule of enrollment, interventions, and assessments is provided in Checklist 1.

Biochemical data will be collected at monthly intervals for the duration of the study, ensuring high-resolution monitoring of disease activity. Elastography will be repeated at predefined intervals, typically every 6 to 12 months or as clinically indicated, to track changes in liver and spleen stiffness over time. Clinical outcomes such as the development of varices, ascites, or hepatic encephalopathy will be monitored continuously through routine clinical care and documented in the study database.

For participants involved in the implementation phase, the timeline includes a randomized crossover simulation in which clinicians will manage deidentified cases under 2 conditions: usual care and CDS-assisted care. This simulation will occur after the development and validation phases are complete. Following the simulation, a pragmatic pilot will be conducted in real-world hepatology clinics, where the CDS tool will be integrated into the EHR and used during routine visits. Clinician usability and trust assessments will be administered at the end of each simulation period and again after the pilot phase.

The overall duration of participation for patients in the observational component is expected to be up to 24 months. Clinicians participating in the implementation evaluation will engage in shorter, structured sessions for simulation and a limited rollout period for the pilot.

### Sample Size and Power Considerations

Because this protocol involves model development, validation, and implementation feasibility, the planned sample size was based on expected cohort availability and anticipated outcome events rather than a conventional hypothesis-testing formula. The sample size determination for this study is primarily driven by the second aim, which focuses on validating the predictive model for CSPH. On the basis of preliminary data and published estimates, we anticipate enrolling approximately 600 patients with PBC across participating sites [7]. This cohort size reflects the expected availability of retrospective and prospective data within the study time frame and ensures adequate representation of key subgroups, including patients with AIH overlap and MASH [19,20].

On the basis of prior PBC cohorts, the prevalence of portal hypertension or CSPH in hepatology referral populations has been reported to range from approximately 24% to 47% depending on disease stage and case mix [25,26]. With a projected sample of 600 participants, this corresponds to an estimated 144 to 282 CSPH events, which provides a sufficient number of outcome events to support model development and validation and evaluate improvements in model discrimination relative to established criteria such as Baveno VII.

In addition, sample size considerations accounted for model complexity. Current methodological guidance for prediction modeling recommends ensuring an adequate number of outcome events relative to the number of predictor parameters to reduce the risk of model overfitting. The anticipated event counts in this cohort are expected to support the planned set of candidate predictors while maintaining an appropriate events-per-predictor ratio for stable model estimation [27].

### Recruitment

Approximately 24 hepatology clinicians will be recruited for the clinician-level randomized crossover simulation. This sample size was selected pragmatically to provide sufficient clinician-level feedback on usability, cognitive workload, trust, and workflow integration while remaining feasible for a pilot implementation study. Eligible clinicians will include hepatologists, advanced practice clinicians, and hepatology trainees with clinical experience managing patients with PBC. Participants will be recruited from hepatology clinics affiliated with academic medical centers.

### Randomization

Randomization will apply only to the implementation phase of the study during the clinician-level crossover simulation. Clinicians will be randomly assigned in a 1:1 ratio to 1 of 2 sequences: usual care followed by CDS-assisted care or CDS-assisted care followed by usual care. A computer-generated randomization schedule will be used to balance allocation between sequences and minimize allocation bias. The simulation will use standardized, deidentified cases rather than live clinical encounters.

**Figure 2.**
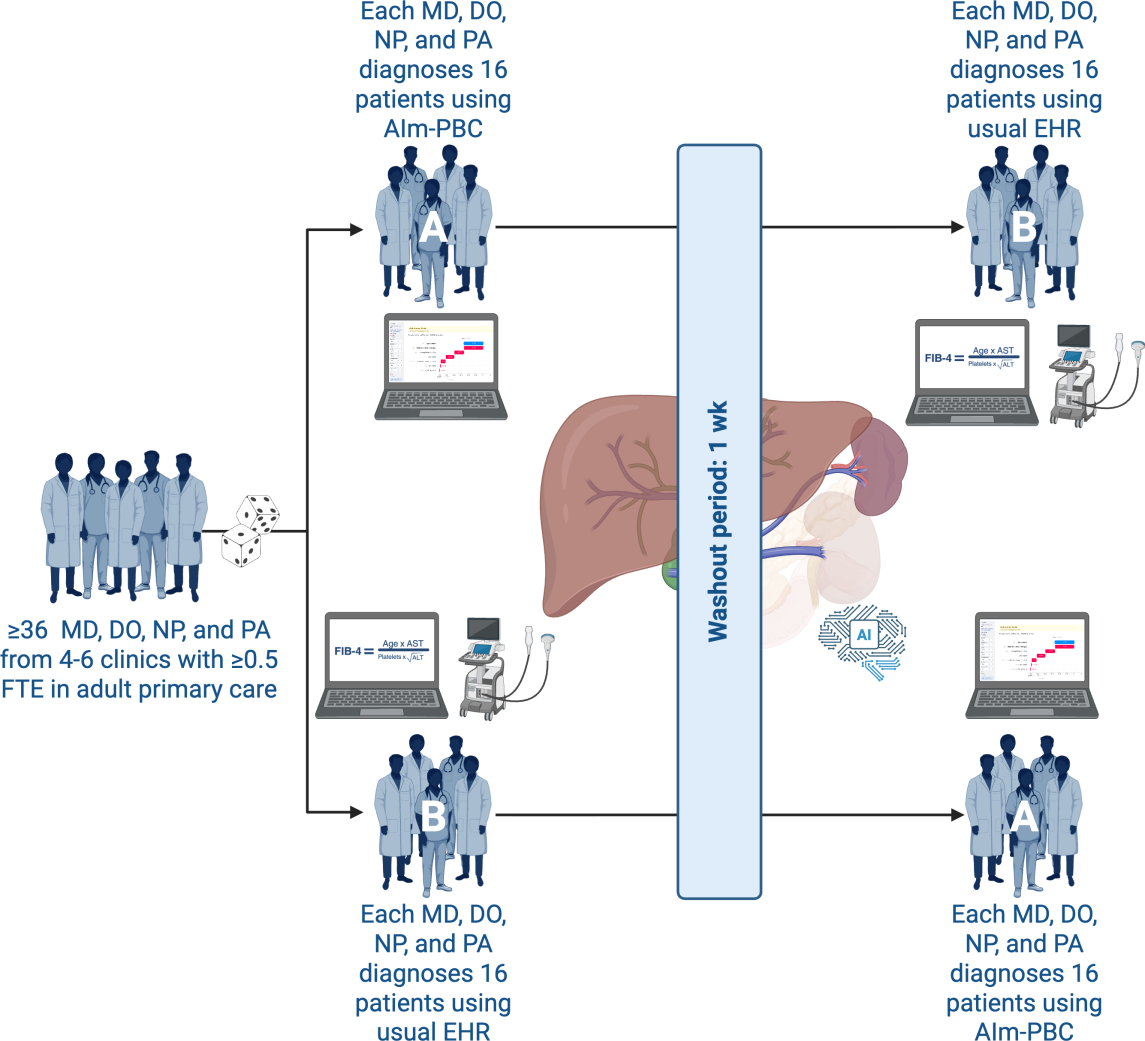
AIm-PBC clinician-level crossover randomized controlled trial pilot design. EHR: electronic health record; FTE: full-time equivalent. DO: doctor of osteopathic medicine; MD: doctor of medicine; NP: nurse practitioner; PA: physician assistant.

To minimize learning and carryover effects, the 2 simulation periods will use distinct, nonoverlapping standardized cases matched in clinical complexity. A 1-week washout period will separate the 2 conditions, and case order will be varied within each period where feasible. Primary comparisons will be conducted within clinicians, allowing each participant to serve as their own control (Figure 2).

### Data Collection

Retrospective data will be sourced from institutional EHRs and data warehouses, capturing historical laboratory values, elastography reports, and clinical outcomes relevant to PBC. Prospective data collection will be conducted in outpatient hepatology clinics using secure, HIPAA (Health Insurance Portability and Accountability Act)-compliant platforms such as REDCap for PROs. These outcomes will include validated instruments such as the WI-NRS, administered twice daily to capture circadian variation in pruritus, and the PBC-40 questionnaire, administered monthly to assess quality of life domains.

Biochemical data, including ALP, bilirubin, aminotransferases, albumin, and platelet count, will be collected at monthly intervals to provide high-resolution longitudinal trajectories. LSM and SSM values will be obtained at baseline and during scheduled follow-up visits, with probe frequency and stiffness values recorded in kilopascals to ensure standardization. Clinical outcomes such as time to variceal development, ascites, and hepatic encephalopathy will be tracked over a 24-month observation period.

All data will be deidentified prior to analysis and stored in secure environments that comply with institutional and federal privacy regulations. Quality control procedures will include automated range checks for laboratory values, standardized reporting templates for elastography, and audit trails for PRO completion. Missing data will be addressed using multiple imputation techniques to minimize bias and preserve statistical power. Data integration will occur through structured pipelines that normalize heterogeneous inputs for use in machine learning models, ensuring consistency across biochemical, imaging, and symptom domains.

### AI Model Development and Validation

The predictive model will be developed using a prespecified machine learning pipeline. Candidate predictors will include longitudinal biochemical markers, platelet count, LSM, SSM, symptom scores, treatment response status, and relevant comorbidities. Features will be selected based on clinical relevance, data availability, and model stability, with highly collinear or sparsely available variables excluded or combined where appropriate.

The dataset will be divided into training, internal validation, and held-out testing subsets at the patient level to avoid data leakage. Model development will use gradient-boosted ensemble algorithms, with hyperparameters tuned within the training set using cross-validation. External validation will be performed using data from at least one participating site not used during model training where feasible.

Class imbalance will be addressed using strategies applied only within the training data, including class weighting or resampling approaches as appropriate. SHAP values will be generated to provide both patient- and cohort-level feature contributions. Model updating will not be performed during initial validation; if performance drift or poor calibration is observed during prospective deployment, recalibration or updating will be conducted using prespecified procedures and reported transparently.

### Statistical Analysis

For the first aim, longitudinal PROs and biochemical markers will be analyzed using mixed-effects models to account for within-subject correlation and repeated measures over time. These models will assess the responsiveness of integrated symptom indexes to changes in biochemical progression or regression, whereas calibration of the AIm-PBC disease activity index will be evaluated against established risk scores such as UK-PBC and GLOBE using the calibration slope, intercept, and Brier score. Discrimination will be quantified through the AUROC, and visualizations of SHAP values will be examined to confirm clinical interpretability.

For the second aim, the predictive model for CSPH complications will be developed using gradient-boosted ensemble algorithms chosen for their robustness in handling heterogeneous data. Model performance will be assessed through the AUROC for discrimination and calibration plots for agreement between predicted and observed outcomes. Clinical utility will be evaluated using decision curve analysis and net reclassification improvement to determine whether the composite model offers meaningful improvement over existing criteria. Comparative analyses will use DeLong tests to assess differences in AUROC between models, and subgroup analyses will be conducted to evaluate fairness and consistency across patients with AIH overlap and MASH. Ablation studies will quantify the incremental value of individual predictors, particularly SSM, by systematically removing variables and observing changes in performance.

For the third aim, implementation outcomes will be analyzed using both descriptive and inferential methods. Usability scores from the System Usability Scale (Multimedia Appendix 1), clinician trust scores from the AI trust scale adapted for AIm-PBC (Multimedia Appendix 2), and cognitive load measures from the National Aeronautics and Space Administration Task Load Index (Multimedia Appendix 3) will be summarized and compared between CDS-assisted and usual care conditions. Decision accuracy and decision time will be analyzed using 2-tailed paired *t* tests within the randomized crossover simulation, whereas clinician adherence to guideline-concordant care pathways will be evaluated using within-clinician comparisons across study phases. Feasibility indicators, such as recruitment and completion rates, will be reported as proportions with CIs. Qualitative debriefing interviews will be analyzed to explore clinician perceptions of usability, workflow integration, trust, and barriers to implementation (Multimedia Appendix 4). Fairness metrics, including AUROC parity gaps across demographic and clinical subgroups, will be calculated to ensure equitable performance of the CDS tool.

All analyses will be conducted using validated statistical software packages in R (R Foundation for Statistical Computing) and Python (Python Software Foundation), with significance thresholds set at a 2-sided α value of .05. Missing data will be addressed through multiple imputation techniques, and sensitivity analyses will be performed to confirm the robustness of the findings.

### Ethical Considerations

Ethics approval for this study will be obtained from the Yale University Human Research Protection Program prior to initiation. The protocol will be registered on ClinicalTrials.gov, and the registration record will be maintained and updated throughout the study to reflect any amendments or changes in status. The approved protocol version number and date will be displayed on all study documents, including the analysis plan, and any site-specific requirements mandated by participating clinics will be honored to ensure compliance with local governance standards.

This study will be conducted across multiple academic centers under a coordinated governance framework. Each participating site will obtain local institutional review board approval or reliance agreements as appropriate. Data sharing arrangements will be governed by formal data use agreements between institutions, ensuring compliance with HIPAA and applicable federal and institutional privacy regulations. All shared datasets will be deidentified prior to transfer and stored in secure, access-controlled environments. All participants will provide written informed consent prior to participation.

A central study steering committee comprising investigators with expertise in hepatology, data science, and implementation science will oversee study conduct, protocol adherence, and data integrity. Algorithm development and validation will be conducted within secure computing environments with version control and audit trails.

During the implementation phase, oversight of the CDS tool will include monitoring for safety, usability, and unintended consequences. Because the CDS tool is intended to support, not replace, clinician decision-making, final clinical decisions will remain at the discretion of the treating clinician. Any unexpected system behavior, performance concerns, or workflow disruptions identified during simulation or pilot deployment will be documented and reviewed by the study team, with predefined procedures for modification or suspension of the tool if necessary.

Any protocol amendments that materially affect eligibility criteria, study outcomes, data collection procedures, statistical analysis methods, consent language, or confidentiality protections will be submitted to the institutional review board for review and approval before implementation. The study team will maintain a comprehensive version history documenting the rationale for each change, along with dates and approvals. When amendments alter information that participants relied upon to provide informed consent, the research team will reconsent all active participants using revised documents. Substantive amendments will also be reflected in the ClinicalTrials.gov record and communicated to site leaders and stakeholders involved in recruitment and oversight.

This protocol has been developed in accordance with the SPIRIT (Standard Protocol Items: Recommendations for Interventional Trials) guidelines (Checklist 1) [28]. A SPIRIT figure outlining the schedule of enrollment, interventions, and assessments is available in Checklist 1. Upon completion, study findings will be disseminated through peer-reviewed publications, presentations at national and international conferences, and summaries shared with participating sites. The final dataset, stripped of identifiers, will be made available through controlled-access repositories under appropriate data sharing agreements to promote reproducibility and secondary analyses. Results will be reported regardless of outcome, and any deviations from the original protocol will be clearly documented and justified in the final manuscript. Additionally, the ClinicalTrials.gov record will be updated with summary results in compliance with federal requirements.

## Results

At the time of manuscript submission, this protocol had not yet begun participant recruitment or data analysis. No participants had been recruited. The study did not receive external funding; therefore, no funding date is applicable. Data collection is projected to begin after institutional review board approval and ClinicalTrials.gov registration, with retrospective data extraction and prospective observational data collection expected to occur over approximately 24 months. The clinician-level randomized crossover simulation and pragmatic pilot implementation phases are expected to occur after model development and validation are completed. Data analysis will begin after completion of the relevant data collection phases. The main study findings are expected to be submitted for publication in summer 2029.

The integrated disease activity index is expected to show strong calibration with established prognostic scores and greater responsiveness to clinically meaningful changes, whereas the inclusion of PROs will provide a more comprehensive view of symptom burden. The predictive model is expected to achieve an AUROC of at least 0.85 for identifying CSPH and deliver a net reclassification improvement of 10% or more over Baveno VII criteria, reducing unnecessary endoscopies without compromising safety. Finally, the CDS prototype is expected to improve clinician adherence to guideline-concordant care pathways, reduce decision time, and achieve high usability and trust scores during the simulation and pilot phases.

## Discussion

### Principal Results

This protocol describes a multiphase study aimed at improving the management of PBC through an explainable AI framework. The anticipated results include a dynamic disease activity index that integrates biochemical, elastography, and patient-reported data, offering superior responsiveness and calibration compared to conventional monitoring. The predictive model is expected to achieve high discrimination for CSPH and outperform existing criteria, reducing unnecessary endoscopies while maintaining patient safety. Finally, the CDS prototype is designed to enhance clinician adherence to guideline-based care, improve decision accuracy, and reduce cognitive load, with usability and trust scores meeting or exceeding established benchmarks.

### Limitations

Several limitations must be acknowledged. The study relies on data from hepatology clinics affiliated with academic centers, which may limit generalizability to community settings. The observational component depends on accurate and complete longitudinal data, and missing data could introduce bias despite planned imputation strategies. Additionally, the implementation phase uses simulated cases before real-world deployment, which may not fully capture the complexity of clinical workflows. Finally, while explainability frameworks are incorporated, clinician adoption of AI tools may still be influenced by factors beyond technical performance, such as institutional culture and resource constraints.

### Comparison With Prior Work

Previous efforts in PBC management have focused primarily on biochemical monitoring and static risk scores such as UK-PBC and GLOBE, which do not account for dynamic symptom burden or integrate noninvasive imaging markers. Similarly, consensus criteria provide useful surrogates for portal hypertension but lack individualized predictive capability. Machine learning models have shown promise in chronic liver disease, yet most operate as opaque systems, limiting clinician trust and adoption. This study builds on these foundations by introducing an explainable, multi-signal approach that combines objective and patient-reported data, integrates noninvasive imaging, and operationalizes insights through EHR-based decision support.

### Conclusions

If successful, this study will demonstrate the feasibility and clinical utility of an explainable AI framework for PBC care, offering a scalable model for precision hepatology. By improving monitoring, risk prediction, and adherence to evidence-based management, the AIm-PBC system has the potential to reduce symptom burden, prevent complications, and enhance quality of life for patients. Future research will focus on external validation across diverse populations and health systems, as well as exploring integration with emerging biomarkers and digital health technologies to further refine predictive accuracy and patient engagement.

## Supplementary material

10.2196/89279 Multimedia Appendix 1 System Usability Scale.

10.2196/89279 Multimedia Appendix 2 AI -Trust Scale (adapted for the AIm-PBC).

10.2196/89279 Multimedia Appendix 3 NASA Task Load Index.

10.2196/89279 Multimedia Appendix 4 Qualitative debriefing interview guide.

10.2196/89279 Checklist 1 SPIRIT (Standard Protocol Items: Recommendations for Interventional Trials) 2025 participant timeline for the AIm-PBC pilot provider-level crossover randomized controlled trial.

## Abbreviations

AI: artificial intelligence
AIH: autoimmune hepatitis
ALP: alkaline phosphatase
AUROC: area under the receiver operating characteristic curve
CDS: clinical decision support
CSPH: clinically significant portal hypertension
EHR: electronic health record
HIPAA: Health Insurance Portability and Accountability Act
LSM: liver stiffness measurement
MASH: metabolic dysfunction–associated steatohepatitis
PBC: primary biliary cholangitis
PRO: patient-reported outcome
REDCap: Research Electronic Data Capture
SHAP: Shapley additive explanations
SMART-on-FIHR: Substitutable Medical Applications, Reusable Technologies on Fast Healthcare Interoperability Resources
SPIRIT: Standard Protocol Items: Recommendations for Interventional Trials
SSM: spleen stiffness measurement
WI-NRS: Worst Itch Numeric Rating Scale
