# Supplementary material for: An Explainable AI Framework for Continuous Monitoring, Risk Stratification, and Clinical Decision Support in Primary Biliary Cholangitis: Protocol for a Multiphase Development and Validation Study
Source: JMIR Res Protoc. 2026 Jun 24;15:e89279. doi: 10.2196/89279 (PMC13294514; doi:10.2196/89279)
Supplement: Multimedia Appendix 1 [file resprot-v15-e89279-s001.docx]

**Multimedia Appendix 1. System Usability Scale (SUS)**

Objective: To measure the perceived usability of the simulated Electronic Health Record interface and the AIm-PBC integration.

Instructions: For each statement, please indicate the degree to which you agree or disagree with the statement.

| **Statement** | **1: Strongly Disagree** | **2: Disagree** | **3: Neutral** | **4: Agree** | **5: Strongly Agree** |
| --- | --- | --- | --- | --- | --- |
| 1. I think that I would like to use this system frequently. | ( ) | ( ) | ( ) | ( ) | ( ) |
| 2. I found the system unnecessarily complex. | ( ) | ( ) | ( ) | ( ) | ( ) |
| 3. I thought the system was easy to use. | ( ) | ( ) | ( ) | ( ) | ( ) |
| 4. I think that I would need the support of a technical person to be able to use this system. | ( ) | ( ) | ( ) | ( ) | ( ) |
| 5. I found the various functions in this system were well integrated. | ( ) | ( ) | ( ) | ( ) | ( ) |
| 6. I thought there was too much inconsistency in this system. | ( ) | ( ) | ( ) | ( ) | ( ) |
| 7. I would imagine that most people would learn to use this system very quickly. | ( ) | ( ) | ( ) | ( ) | ( ) |
| 8. I found the system very cumbersome to use. | ( ) | ( ) | ( ) | ( ) | ( ) |
| 9. I felt very confident using the system. | ( ) | ( ) | ( ) | ( ) | ( ) |
| 10. I needed to learn a lot of things before I could get going with this system. | ( ) | ( ) | ( ) | ( ) | ( ) |
