# Supplementary material for: An Explainable AI Framework for Continuous Monitoring, Risk Stratification, and Clinical Decision Support in Primary Biliary Cholangitis: Protocol for a Multiphase Development and Validation Study
Source: JMIR Res Protoc. 2026 Jun 24;15:e89279. doi: 10.2196/89279 (PMC13294514; doi:10.2196/89279)
Supplement: Multimedia Appendix 2 [file resprot-v15-e89279-s002.docx]

**Multimedia Appendix 2. AI-Trust Scale (Adapted for AIm-PBC)**

Objective: To measure the provider's trust and confidence in the AIm-PBC decision-support tool.

Instructions: For each statement regarding the AIm-PBC tool, please indicate the degree to which you agree or disagree. This survey is only completed after the AIm-PBC-Enabled Care (Condition A) period.

| **Statement** | **1: Strongly Disagree** | **2: Disagree** | **3: Neutral** | **4: Agree** | **5: Strongly Agree** |
| --- | --- | --- | --- | --- | --- |
| 1. I trust the risk prediction for clinically significant portal hypertension provided by AIm-PBC. | ( ) | ( ) | ( ) | ( ) | ( ) |
| 2. AIm-PBC appears reliable in correctly identifying patients who require referral/VCTE. | ( ) | ( ) | ( ) | ( ) | ( ) |
| 3. The Shapley Additive Explanations (SHAP) panel helped me understand why the score was generated. | ( ) | ( ) | ( ) | ( ) | ( ) |
| 4. The explanation panel made me more confident in the triage recommendation. | ( ) | ( ) | ( ) | ( ) | ( ) |
| 5. I would be comfortable explaining the AIm-PBC results to a patient. | ( ) | ( ) | ( ) | ( ) | ( ) |
| 6. I believe the AIm-PBC risk estimate is free of bias against patient subgroups (e.g., age, sex, race). | ( ) | ( ) | ( ) | ( ) | ( ) |
| 7. I believe AIm-PBC is more precise than traditional scores (e.g., platelet count, liver stiffness, spleen stiffness, albumin, FIB-4) for managing PBC risk. | ( ) | ( ) | ( ) | ( ) | ( ) |
