# Supplementary material for: An Explainable AI Framework for Continuous Monitoring, Risk Stratification, and Clinical Decision Support in Primary Biliary Cholangitis: Protocol for a Multiphase Development and Validation Study
Source: JMIR Res Protoc. 2026 Jun 24;15:e89279. doi: 10.2196/89279 (PMC13294514; doi:10.2196/89279)
Supplement: Multimedia Appendix 3 [file resprot-v15-e89279-s003.docx]

**Multimedia Appendix 3. NASA Task Load Index (NASA-TLX)**

Objective: To assess the cognitive workload experienced by the provider during the simulated cases for both the usual care and AIm-PBC-enabled conditions.

Instructions: Please rate the case simulation period based on the following six factors.

| **Dimension** | **Description** | **Low (1)** | **High (5)** |
| --- | --- | --- | --- |
| **1. Mental Demand** | How much mental and perceptual activity was required (e.g., thinking, deciding, calculating, remembering)? | Very Low | Very High |
| **2. Physical Demand** | How much physical activity was required (e.g., pushing, pulling, turning, controlling, activating)? | Very Low | Very High |
| **3. Temporal Demand** | How much time pressure did you feel due to the pace at which the tasks or task elements occurred? | Very Low | Very High |
| **4. Performance** | How successful were you in accomplishing the goals of the task, and how satisfied were you with your performance? | Perfect | Failure |
| **5. Effort** | How hard did you have to work (mentally and physically) to achieve your level of performance? | Very Low | Very High |
| **6. Frustration** | How insecure, discouraged, irritated, or stressed were you vs. secure, gratified, content, and relaxed? | Very Low | Very High |
