# Supplementary material for: An Explainable AI Framework for Continuous Monitoring, Risk Stratification, and Clinical Decision Support in Primary Biliary Cholangitis: Protocol for a Multiphase Development and Validation Study
Source: JMIR Res Protoc. 2026 Jun 24;15:e89279. doi: 10.2196/89279 (PMC13294514; doi:10.2196/89279)
Supplement: Multimedia Appendix 4 [file resprot-v15-e89279-s004.docx]

**Multimedia Appendix 4. Qualitative Debrief Interview Guide**

Objective: To explore barriers, facilitators, and perceptions of fairness and clinical utility, administered after the provider completes both periods (T4).

Instructions: This is a semi-structured interview. The interviewer will use these questions as a guide but will allow for participant-driven discussion.

***A. Implementation and Workflow***

1. Describe how the AIm-PBC tool (Condition A) did or did not change your typical workflow when evaluating PBC risk factors.
2. Were the decision times you experienced (Target: minutes) generally acceptable for a primary care setting? What contributed most to efficiency or delay?
3. How did the integrated features in the EHR (e.g., SmartPhrases, pre-built order sets) support or hinder the adoption of the AIm-PBC recommendations?
4. What were the most significant challenges you encountered while using the AIm-PBC interface?

***B. Trust, Transparency, and Decision-Making***

1. How did the Shapley Additive Explanations (SHAP) panel influence your confidence in the AIm-PBC score compared to simply seeing the score alone?
2. Were there any cases where your clinical judgment conflicted with the AIm-PBC recommendation? If yes, what factors led you to override the recommendation, or consider overriding it?
3. In your opinion, what is the most compelling feature of AIm-PBC that would drive its adoption into live clinical practice?

***C. Fairness and General Feedback***

1. In the simulated cases, did you notice any trends or concerns regarding how AIm-PBC performed for specific patient demographic subgroups (e.g., older vs. younger patients, different races/ethnicities, or BMI)?
2. If this tool were rolled out tomorrow, what is the single most important piece of information you would want the developers or your colleagues to know about it?
3. Do you feel your participation in this simulated trial adequately prepared you to provide feedback on the real-world utility of AIm-PBC?
