# Supplementary material for: An Explainable AI Framework for Continuous Monitoring, Risk Stratification, and Clinical Decision Support in Primary Biliary Cholangitis: Protocol for a Multiphase Development and Validation Study
Source: JMIR Res Protoc. 2026 Jun 24;15:e89279. doi: 10.2196/89279 (PMC13294514; doi:10.2196/89279)
Supplement: Checklist 1 [file resprot-v15-e89279-s005.docx]

**SPIRIT 2025 Participant Timeline for the AIm-PBC Pilot Provider-Level Crossover Randomized Controlled Trial**

| **DOMAIN** | **Procedure** | **Enrollment** | **Allocation** | **Intervention Period 1** | **Washout** | **Intervention Period 2** | **Close-out** |
| --- | --- | --- | --- | --- | --- | --- | --- |
|  | **Timepoint** | **T-2 weeks to T0** | **T0** | **T1 (1-2 weeks)** | **T2 (1 week)** | **T3 (1-2 weeks)** | **T4** |
| **ENROLLMENT** |  |  |  |  |  |  |  |
| Eligibility Screen & Recruitment |  | X |  |  |  |  |  |
| Informed Consent |  | X |  |  |  |  |  |
| Orientation & Technical Competency Check |  | X |  |  |  |  |  |
| **ALLOCATION** |  |  |  |  |  |  |  |
| Randomization to Sequence (AB or BA) |  |  | X |  |  |  |  |
| **INTERVENTION** |  |  |  |  |  |  |  |
| AIm-PBC-Enabled Care (Condition A) |  |  |  | X (in Sequence AB) |  | X (in Sequence BA) |  |
| Usual Care (Condition B) |  |  |  | X (in Sequence BA) |  | X (in Sequence AB) |  |
| **ASSESSMENTS** |  |  |  |  |  |  |  |
| **Feasibility Outcomes** |  |  |  |  |  |  |  |
| Decision Time (Workflow Efficiency) |  |  |  | X |  | X |  |
| **Usability** (SUS Score) |  |  |  | X |  | X |  |
| **Trust in AI** (AI-Trust Scale) |  |  |  | X |  | X |  |
| **Cognitive Workload** (NASA-TLX) |  |  |  | X |  | X |  |
| **Effectiveness Outcome** |  |  |  |  |  |  |  |
| Diagnostic Accuracy (Sensitivity, Specificity, AUROC) |  |  |  | X |  | X |  |
| **Secondary Outcomes** |  |  |  |  |  |  |  |
| Referral Rates, Confidence, Override Rates |  |  |  | X |  | X |  |
| Qualitative Debrief Interview (Barriers/Facilitators) |  |  |  |  |  |  | X |
| Final Data Verification & Feedback |  |  |  |  |  |  | X |
